# Technical note: No increase in effective dose from half compared to full rotation pelvis cone beam CT

**DOI:** 10.1002/acm2.12150

**Published:** 2017-08-02

**Authors:** Pascal Hauri, Roger A. Hälg, Uwe Schneider

**Affiliations:** ^1^ Department of Physics University of Zurich Zurich Switzerland; ^2^ Radiotherapy Hirslanden Hirslanden Medical Center Aarau Switzerland

**Keywords:** abdomen, Cone Beam CT, effective dose, radiation therapy

## Abstract

**Purpose:**

To image the abdomen of a patient with a gantry mounted imaging system of a linear accelerator, different cone beam computed tomography (CBCT) protocols are available. The whole‐body dose of a full rotation abdomen CBCT and a half rotation CBCT was compared. In our clinic, both CBCT protocols are used in daily routine work.

**Methods:**

With an adult anthropomorphic Alderson phantom, the whole‐body dose per CBCT scan was measured with thermoluminescence dosimeters. The half rotation CBCT was applied such that the gantry mounted X‐ray source rotated around the right side of the phantom. The 183 measurement locations covered all ICRP recommended critical organs (except the gonads). The effective dose was calculated with the mean organ dose and the corresponding tissue weighting factors. A point‐by‐point dose comparison of both protocols was conducted.

**Results:**

The effective dose was 5.4 mSv ±5% and 5.0 mSv ±5% (estimated type B 1*σ*) for the full and the half rotation CBCT respectively. There was no significant difference (*α* = 0.05) in the effective dose within the precision of the measurement (1*σ* = 5%). The half rotation CBCT displayed an inhomogeneous dose distribution in a transversal phantom slice in contrast with the full rotation CBCT. In the imaging region, the mean dose was (20.5 ± 3.4) mGy and (19.2 ± 7.4) mGy (measured type A 1*σ*) for the full and the half rotation CBCT respectively.

**Conclusion:**

The half compared to the full rotation CBCT displays a smaller field‐of‐view in a transversal slice and no significant difference in the effective dose. Hence, the full rotation CBCT is favorable compared to the half rotation CBCT. However, by using the half rotation protocol, critical volumes in the patient can be spared compared to the full rotation protocol.

## 1 INTRODUCTION

Cone beam computed tomography (CBCT) is widely used in clinics for patient positioning in a radiation treatment session. The advantage of image‐guided radiation therapy (IGRT) is the application of more conformal plans and therefore, a reduction of the irradiated volume. The additional dose received by the patient^1^ raises concerns about late effects such as second primary cancers. Hence, the quantification of CBCT dose is an important issue.

The TrueBeam linear accelerator (Varian Medical Systems, Palo Alto, CA, USA) is equipped with a gantry mounted on‐board imager (OBI) capable of performing CBCT scans. To image the abdomen, the pelvis or the pelvis spotlight protocol is available. For the pelvis protocol, the CBCT is acquired with a full rotation of the X‐ray source around the patient. The pelvis spotlight protocol uses a half rotation of the X‐ray source around the patient. The weighted CT dose index (CTDI_*w*_) given by Varian, is 1/3 lower for the half compared to the full rotation CBCT. Hence in clinical practice, the spotlight protocol is used more frequently because of the potential dose reduction compared to the full rotation protocol. The resulting spotlight CBCT has a smaller field‐of‐view in a transversal slice compared to the full rotation protocol.

In the last years, many studies have been conducted about CBCT dose for different protocols.^2^, ^3^, ^4^, ^5^, ^6^, ^7^, ^8^, ^9^ Some of them evaluated only the CTDI values.^3^, ^9^ Kan et al.,^10^ Cheng et al.,^6^ and Hälg et al.^7^ determined the effective dose (ED) for pelvis protocols using the OBI of a Varian linear accelerator. In another paper,^2^ Monte Carlo (M.C.) dose calculations of different pelvis CBCT protocols were performed for real patient geometries.

With the OBI (version 2.5.28.0) of a TrueBeam linear accelerator, the absorbed dose for the latest pelvis and pelvis spotlight CBCT protocol was measured. Both CBCT protocols are used in‐house in daily routine work. The 183 measurement locations in an anthropomorphic Alderson phantom were equipped with thermoluminescence dosimeters (TLDs). A combination of Li:Mg,Ti (TLD100) and Li:Mg,Cu,P (TLD100H) chips was used to automatically correct for the variation in response with radiation energy of the TLDs.^11^ This allowed a more accurate determination of the whole‐body absorbed dose (1*σ* = ±5%) compared to previous studies. The ED values of both pelvis protocols were calculated by the determination of the mean doses to critical structures and the ICRP guidelines.^12^ Furthermore, the absorbed dose of the pelvis and the pelvis spotlight CBCT was compared on a point‐by‐point basis.

## 2 METHODS AND MATERIALS

All measurements and detector readouts were performed according to a strict protocol to ensure the consistency of the measurements.

### 2.A Imaging modality and whole‐body dose measurement

The two evaluated CBCT pelvis protocols were provided by the vendor (see Table 1). For both protocols the X‐ray source was operated at 125 kVp, which resulted in a mean photon energy of 64 keV.^9^ The CBCT measurements were done at Hirslanden Medical Center in Aarau, Switzerland.

**Table 1:** Acquisition parameters for the two different kV CBCT protocols given by Varian (version 2.5.28.0). In our clinic, both protocols are used in daily routine work in the current form

	Pelvis	Pelvis spotlight
Peak voltage (kVp)	125	125
Exposure (mAs)	1080	750
Fan type	Half	Full
Gantry rotation (degree)	360°	209°
(up,left,down,right) = (0°,90°,180°,270°)	180°↔180°	256°↔105°
Field‐of‐view diameter (cm)	46.5	26.2
Transversal dimension from isocenter (cm)	±8.75	±9.25
Slice thickness (mm)	2.0	2.0
Matrix (pixel)	512	512
Projections	900	500
CTDI_w_ (mGy)	14.3	10.1

The absorbed dose of the CBCTs was measured using an adult anthropomorphic Alderson‐Rando phantom (RSD Radiology Support Devices, Long Beach, CA, USA). The phantom was positioned head first supine. The measurement locations were distributed in the Alderson phantom according to Hälg et al.^7^ The locations covered the ICRP recommended critical organs (except the gonads). At each of the 183 measurement location in and on the phantom, a TLD100H was stacked on top of a TLD100 for dose measurement. To have a dose of at least 1 mGy at each measurement location, the full and the half rotation CBCT was irradiated 45 and 55 times respectively. The dose of each CBCT protocol was normalized to one scan. The dose of 1 mGy ensured enough signal for the readout of the TLDs. The pelvis spotlight CBCT was acquired by rotating the cone beam over the right side of the Alderson phantom [see Table 1 and Fig. 2(b)].

### 2.B TLD dose evaluation

The whole‐body dose of the two CBCT protocols was measured with a combination of TLD100 and TLD100H chips. The two TLD types show a different response with radiation energy compare to ^*60*^Co.^13^ For the dose measurements, a TLD100H chip was put on top of a TLD100 chip. The doses measured by the TLD100 and TLD100H were evaluated by using individual calibration factors determined with a 6 MV nominal beam energy irradiation applied with a TrueBeam linear accelerator. The individual energy correction factor for TLD100 and TLD100H was found by the ratio of the TLD100 divided by the TLD100H dose. Hence, each single TLD was corrected with a specific energy correction factor displaying a random error. The finale dose was calculated with the mean of the corrected TLD100 and TLD100H dose. A detailed description of the TLD dose and energy measurement is given by Hauri et al.^11^ All absolute dose measurements were correlated to a Farmer Chamber 30013 (PTW, Freiburg, Germany).

### 2.C Dose comparison

ED values were obtained by using the tissue weighting factors from ICRP publication 103^12^ and the mean organ doses. Each organ dose was calculated by the mean of the determined point doses at the corresponding organ location. The image isocenter was located in the prostate region. The gonad dose had to be estimated due to missing measurement points. For this the mean of six dose points was calculated. The points were located in a transversal slice 5 cm from the image isocenter in direction “feet toward head” (FH). In this transversal phantom slice, the points to estimate the gonad dose were located in the right to left (RL) middle of the phantom, along a line anterior posterior. The gonad location in the phantom was at 5 cm from the image isocenter in the FH direction. The ED was calculated as according to the ICRP 103^12^ recommendation:

(1) $$\mathrm{ED}=\sum_{T}w_{T}H_{T}=\sum_{T}w_{T}\sum_{R}w_{R}D_{T,R}.$$

where $w_{R}$ is the radiation weighting factor with $w_{R}=1$ for photon irradiations. $D_{T,R}$ is the mean absorbed dose from radiation R in tissue T. $w_{T}$ is the tissue weighting factor for tissue T with $\sum_{T}w_{T}=1$. $H_{T}=\sum_{R}w_{R}D_{T,R}$ is the equivalent dose for tissue T. Furthermore, the absorbed dose of the two different CBCT protocols was evaluated by a point‐by‐point comparison.

## 3 RESULTS

The estimated type B standard deviation (1*σ*) for a CBCT dose point measurement was ±5%. A more detailed error analysis can be found in Hauri et al.^11^

In Table 2 the effective organ doses are displayed for both CBCT protocols. Furthermore, the number of measurement points to calculate the mean organ dose is shown. The ED was determined to 5.4 mSv ±5% and 5.0 mSv ±5% (type B 1*σ*) for the full and the half rotation CBCT respectively. The 95% confidence interval of the difference between the two ED values contained zero (0.4 ± 0.7 mSv). Hence, there was no significant difference (*α* = 0.05) of the ED values between the two protocols within the precision of the measurement (±5%).

**Table 2:** The ICRP weighting factors wT^12^ and the determined equivalent dose HT of the organ T for both CBCT protocols (OBI version 2.5.28.0). Furthermore, the number of measurement points to calculate the mean absorbed organ dose is shown. The mean red bone marrow and bone surface doses were determined from the same 33 measurement locations. The effective dose for the pelvis and the pelvis spotlight was calculated to (5.4 ± 0.3) mSv and (5.0 ± 0.3) mSv respectively

	*w*_*T*_	*H*_*T*_ pelvis (mSv)	*H*_*T*_ pelvis spotlight (mSv)	Number of measurement points
Gonads	0.08	23.8	22.0	6
Red bone marrow	0.12	4.49	4.31	33
Colon	0.12	6.09	5.01	17
Lung	0.12	0.190	0.154	30
Stomach	0.12	0.277	0.209	4
Breasts	0.12	0.215	0.201	4
Bladder	0.04	20.0	18.4	2
Liver	0.04	0.468	0.511	11
Esophagus	0.04	0.135	0.119	5
Thyroid	0.04	0.0875	0.0716	2
Skin	0.01	17.7	17.1	5
Bone surface	0.01	4.49	4.31	33
Salivary glands	0.01	0.0695	0.0652	4
Brain	0.01	0.0526	0.0451	10
Remainder of body	0.12	9.23	8.62	56

**Figure 1:**
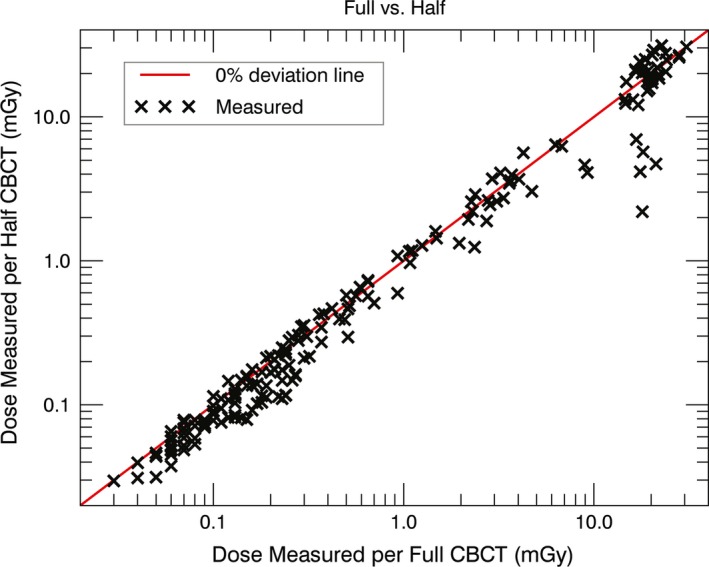
Measured point doses per CBCT scan in the Alderson phantom. Each cross corresponds to a unique measurement location for the pelvis and the pelvis spotlight protocol. For all points below the 45° line the dose of the pelvis spotlight was lower compared to the pelvis protocol.

Figure 1 shows the point‐by‐point comparison of the absorbed dose for both CBCT protocols. The highest dose per scan was located in the field‐of‐view region. Here, the biggest differences in dose between the full and the half rotation CBCT was present. However, the mean dose of 37 measurement locations in the field‐of‐view region was 20.5 ± 3.4 mGy and 19.2 ± 7.4 mGy (measured type A 1*σ*) for the full and the half rotation CBCT respectively. Outside the image region both protocols showed a similar dose per scan (Fig. 1). The peripheral dose decreased from 15 to 0.030 mGy with increasing distance to the scanned region.

**Figure 2:**
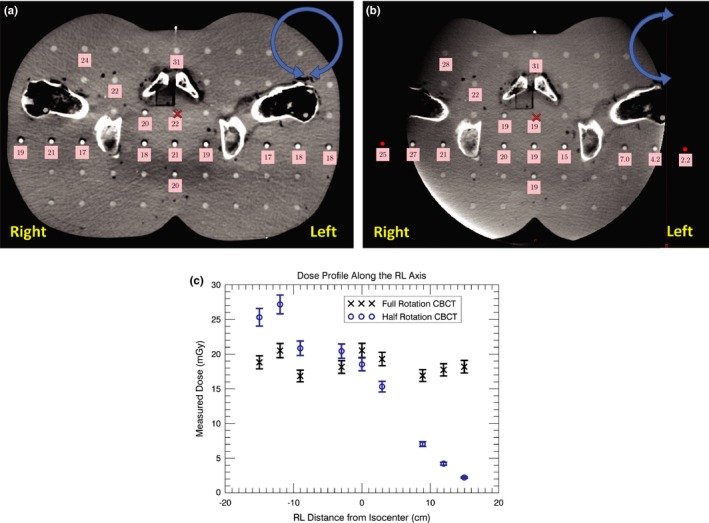
The measured point doses (mGy) per scan in a transversal slice of the Alderson phantom for (a) the pelvis CBCT and (b) the pelvis spotlight CBCT. The arrow in the right upper corner of the CBCT slice indicates the rotation of X‐ray source around the phantom. The cross in the milled of the slice represents the image isocenter. (c) The dose profiles along the RL axis of the full and the half rotation CBCT for the same transversal slice from (a) and (b). The error bars represent the standard deviation of the dose measurements.

Figures 2(a) and 2(b) show the dose in the same transversal CBCT slice in the imaging region for the full and half rotation CBCT respectively. The dose of the full rotation CBCT was homogeneously distributed in a transversal slice of the phantom. In the RL middle of the slice, the dose of both protocols was similar. On the right side of the phantom, the half rotation CBCT displayed a higher dose compared to the full rotation CBCT. In contrast, on the phantoms left side the dose of the spotlight CBCT was lower compared to the pelvis CBCT.

For the half rotation CBCT, the dose in the phantoms right side was higher compared to the left side. In the scanned region, the dose was up to a factor of ten higher on the right compared to the left side of the phantom [Figs. 2(b) and 2(c)]. Outside the imaging region, the dose on the right side was a factor of two higher compared to the phantom's left side. The right eye measurement location displayed 0.09 mGy per spotlight scan while for the left eye a dose of 0.05 mGy was noticed. The dose of the left eye for the full rotation CBCT was 0.08 mGy.

## 4 DISCUSSION

With the OBI of a TrueBeam Varian linear accelerator, a full rotation and a half rotation CBCT protocol (Table 1) were measured. Both CBCT protocols are used in daily routine work. At 183 points in and on an anthropomorphic Alderson phantom the dose per scan was evaluated. For both protocols the ED was calculated and the absorbed dose was compared on a point‐by‐point basis. Both ED values were in the range of 5 mSv. Within the precision of the measurement (type B 1*σ* = ±5%), there was no significant difference (*α *= 0.05) between the two ED values. An inhomogeneous dose deposition in transversal phantom slices was noticed for the spotlight CBCT protocol.

Despite the efforts to reduce the dose to the patient, the H_T_ and ED values determined by others in 2011 (OBI version 1.4.13)^6^ and 2012^7^ are of the same order as presented in this study. Hälg et al.^7^ used the same measurement location and the same phantom as in the current study. Nevertheless, in a study from 2008^10^ a four times higher ED was reported for the Varian system. The mean organ doses determined by M.C. simulations for real patient geometries 2 are in the same range as presented here (Table 2). Hence, the anthropomorphic phantom used for the measurements represents an adult patient geometry well. The determined CBCT dose for an adult should not be projected to a pediatric patient, since the absorbed dose in a young patient is likely to be higher.^8^ The higher dose is caused by less attenuation of the X‐ray beam in the pediatric compared to the adult patient. Furthermore, in an adolescent patient critical structures are closer to the imaging region compared to an adult patients.

In the image region, a dose reduction by a factor of ten for critical points can be achieved by using the half compared to the full rotation CBCT. To accomplish a dose reduction for a critical volume, the starting angle of the half rotation CBCT acquisition is crucial [see Fig. 2(b)]. Nevertheless, there is no significant reduction in ED for the half compared to the full rotation CBCT.

According to Gardner et al.,^4^ the contrast‐to‐noise ratio of the pelvis compared to the spotlight protocol is significantly higher. Hence, the full rotation CBCT provides better soft tissue contrast compared to the half rotation protocol.

The ED is a function of the mean organ dose. Therefore, the ED values depend on the distribution of the measurement location in the phantom. This study represents a conservative estimation of the ED since 1/5 of the TLDs were distributed in the imaging region representing 1/10 of the body. The type B uncertainty of the dose measurement (1*σ* = ±5%) was propagated to the ED. This is rather an overestimation of the ED uncertainty since H_T_ was calculated by the mean of multiple measurement locations (Table 2).

The higher photoelectric crosssection of bones compared to tissue is reported to cause a few times higher dose to bones then to nearby tissue.^2^, ^5^ For the TLDs distributed in the human bony material of the Alderson phantom used in this work, no higher dose was notice compared to the surrounding soft tissue. Hence, the equivalent dose to bones could be underestimated.

## CONCLUSION

The half compared to the full rotation CBCT displays a smaller field‐of‐view and no significant difference in the ED. Hence, the full rotation CBCT is favorable compared to the half rotation CBCT. However, by using the half rotation protocol critical points in the patient can be spared compared to the full rotation protocol.

## CONFLICT OF INTEREST

The authors declare no conflict of interest.
